# Impact of Molybdenum Compounds as Anticancer Agents

**DOI:** 10.1155/2019/6416198

**Published:** 2019-09-10

**Authors:** Ayodele T. Odularu, Peter A. Ajibade, Johannes Z. Mbese

**Affiliations:** ^1^Department of Chemistry, University of Fort Hare, Private Bag X1314, Alice 5700, South Africa; ^2^School of Chemistry and Physics, University of KwaZulu-Natal, Pietermaritzburg Campus, Scottsville 3209, South Africa

## Abstract

The aim of this mini review was to report the molybdenum compound intervention to control cancer disease. The intervention explains its roles and progress from inorganic molybdenum compounds via organomolybdenum complexes to its nanoparticles to control oesophageal cancer and breast cancer as case studies. Main contributions of molybdenum compounds as anticancer agents could be observed in their nanofibrous support with suitable physicochemical properties, combination therapy, and biosensors (biomarkers). Recent areas in anticancer drug design, which entail the uses of selected targets, were also surveyed and proposed.

## 1. Introduction

The increase in the population of those affected with cancer globally warrants a swift attention [1–6]. The increase might be due to aging, world population growth, and cancer-causing behaviours (smoking) [6]. From Jemal et al.'s study, they predicted more in the increase of all-cancer cases from 12.7 million emergent cases in 2008 to 22.2 million by 2030 [6].

Drugs used to control the increasing cancer are either cytotoxic (cell killing) or cytostatic (cell stabilizing) [1]. Both drugs lead to decrease in the tumour size due to the reason that cancer cells have a high mortality rate which stops them from splitting up, thereby resulting in a decrease in their populace [1]. In order to further bring decrease in the populace, the global research is focusing on the development of existing agents and the innovation of novel biological objectives. Medicinal application of metals can be dated back to five thousand years ago [1]. Metalloelements in trace amounts execute important activities in the living system. Among these metalloenzymes are transition metals, which represent the d block elements between Groups 3 and 12 in the periodic table [1]. The d block transition metals in the periodic table have partially filled electrons (d orbitals), which influence remarkable electronic properties when examined for the development and design of anticancer agents. This property of transition metals leads to the foundation of coordination compounds (metal complexes) [1]. The transition metal ions play important roles in appropriate functioning of various enzymes. The ligation of different bioligands to biometals enhances the metals' activities. The ability of ligands to coordinate metals in a three-dimensional arrangement permits groups' functionalization which can be designed to distinct molecular targets in the development of novel medicinal agents. The mode of biological action for coordination compounds relies on the thermodynamic and kinetic properties. The drug's lipophilicity is improved through chelates' formation, and drug action is extensively enhanced because of efficient drug permeability into the site of action. Additionally, coordination compounds implement important activities in agriculture and pharmaceutical industries.

In 1960, an antitumour action of an inorganic compound of cis-diammine-dichloroplatinum(III) (cisplatin) was discovered [1]. Today, in the clinics, cisplatin (platinum metal ammine) is a global cytotoxic drug for cancer treatment [1]. Further development of cisplatin had made it the most efficient drug for solid carcinomas' treatment.

In order to improve the global anticancer study, comes the question, what impact does molybdenum play?

Molybdenum has been known to exist in history. Carl Wilhelm Scheele discovered the element in 1778, while Peter Jacob Hjelm first isolated it in 1781 [7]. Molybdenum is the least abundant element in Group VI. It is extensively distributed in nature. The natural occurrence in the combined state most often occurs as ores of molybdenite (MoS_2_) and wulfenite (PbMoO_4_). It is silvery white in appearance and belongs to the chromium group.

Molybdenum is a second-row transition element [8, 9]. It has a symbol of Mo, atomic number of 42, mass number of 95.94, and electron configuration of [Kr] 4d^5^5s^1^. It has a range of oxidation states from +2 to +6, that is, has five valances (+2, +3, +4, +5, and +6), where oxidation states from +2 to +5 are air sensitive. The key isotopes are ^95^Mo, ^96^Mo, and ^98^Mo.

Salts of molybdenum with oxidation states ranging from +3 to +6 can be formed with the exception of +5. Mo(VI) salts are the most stable. It forms stable and water-soluble compounds in trivalent and hexavalent states.

The hardness, durability, and toughness make them essential alloys and steels. Apart from the use of molybdenum as alloys and steels, other industrial uses include the use of molybdenum sulfide as a lubricant, colorant for ceramic and textiles, building aircraft and missile parts, application in nuclear energy, filaments in electrical devices, building filament supports in incandescent lamps, and electrodes for electrically heated glass furnaces. It is used industrially as a catalyst to refine petroleum. On the other hand, Mo can also be found in different concentrations in water.

Molybdenum is a vital trace element for humans, animals, and plants. It serves as an essential trace element in the nutrition of plants. Molybdenum in trace concentration can be found in plant-derived foods like cereal grains, cheese, leafy vegetables, legumes, milk, nuts, and organ meats. This depends on the concentration of molybdenum present in the soil of the growing region. In the human body, molybdenum is stored in the bones, glands, liver, and kidneys. It can also be located in the lungs, muscles, skin, and spleen, but almost 90% of molybdenum eaten from foods is eradicated from digested foods through the urine. Medical applications of molybdenum are numerous including avoidance of dental caries, cure of anaemia, enhancement of immunological reactions, as *anticancer* and antidiabetic agents. Molybdenum has an antagonistic action against copper; that is, high concentrations of molybdenum can decrease copper absorption and afterwards cause copper deficiency [7].

Molybdenum atoms go through transition between oxidation states of IV and VI, during enzymatic reactions [10]. Molybdenum, as a constituent of molybdoprotein, participates to form active sites for numerous enzymes. The three main molybdenum-containing enzymes are aldehyde oxidase, dehydrogenase/oxidase, and sulphite oxidase. Molybdenum containing enzymes perform three functions, namely, purine catabolism, protein synthesis stimulation, and body growth [7].

Fisher et al. reported the ambiguous impact of molybdenum in xanthine oxidase (XOD (flavoprotein enzyme)), while Hille et al. stated that XOD was the first evidence of the biological relevance of molybdenum in molybdenum-consisting metalloenzymes [11, 12].

Healthwise, Chan et al. [13] and Dmedley et al. [14] explained that exposure to the element can be detrimental, with scarce indication for signs in humans. Komada et al. reported that low molybdenum content in South African and China soils resulted in esophageal cancer [15]. Nouri et al.'s works were in line with those of Komada et al. when they reported low and moderate occurrence of esophageal cancer in Iran soils was due to low molybdenum content [16]. Researchers had reported the biological application of molybdenum as antibacterial, *anticancer*, antifungal, and antiulcer agents [17–20]. The motivation for opting for molybdenum among several metals was due to its wide labile chemistry and low toxicity [21, 22]. This review aimed to report on the advances in the use of molybdenum compounds as anticancer agents against esophageal cancer and breast cancer. Esophageal cancer and breast cancer were looked into among other types of cancer diseases because food has to pass through the esophageal (gullet) for it to be digested and provide energy, while babies feed on milk from women's breast for the first few months of the growth.

## 2. Molybdenum Compounds and Molybdenum Complexes as Anticancer Agents in Chemotherapy

The current treatments for cancer are surgery, radiation, and chemotherapy.

**Figure 1:**
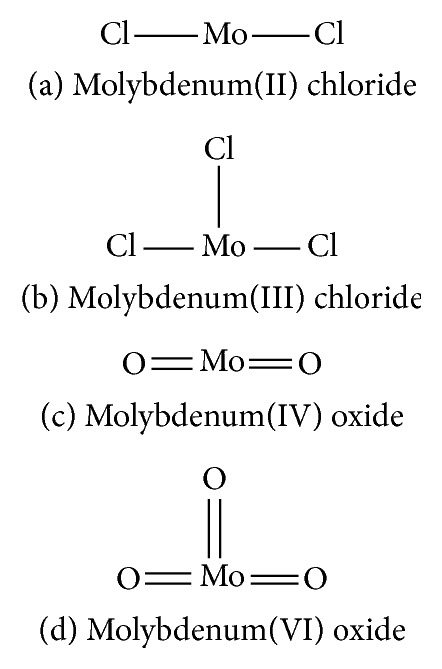
Inorganic molybdenum compounds. (a) Molybdenum(II) chloride. (b) Molybdenum(III) chloride. (c) Molybdenum(IV) oxide. (d) Molybdenum(VI) oxide.

In chemotherapy, certain classifications of significant inorganic compounds (molybdenum halides (molybdenum(II) chloride and molybdenum(III) chloride), molybdenum oxides (molybdenum(IV) oxide (MoO_2_), and molybdenum(VI) oxide (MoO_3_)), iso- and hetero-polyoxomolybdates), molybdenum hexacarbonyl and hybrid inorganic-organic materials, and molybdenum oxides (Mo_*n*_W_1−*n*_O_3_), with chemical structures are shown in Figure 1. They are used vastly for medicinal applications [23, 24].

**Figure 2:**
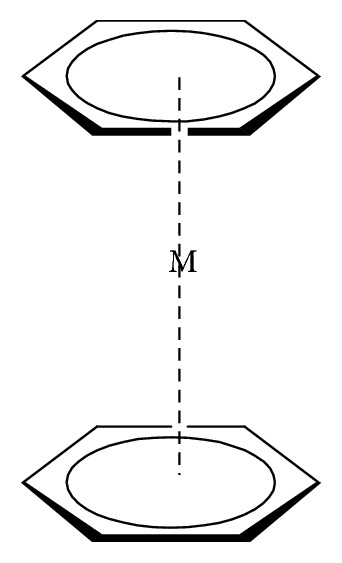
Metallocene.

**Figure 3:**
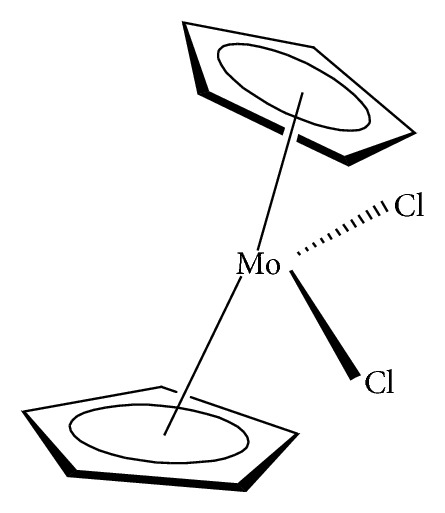
Dichlorobismolybdenum(IV) (molybdocene dichloride).

**Figure 4:**
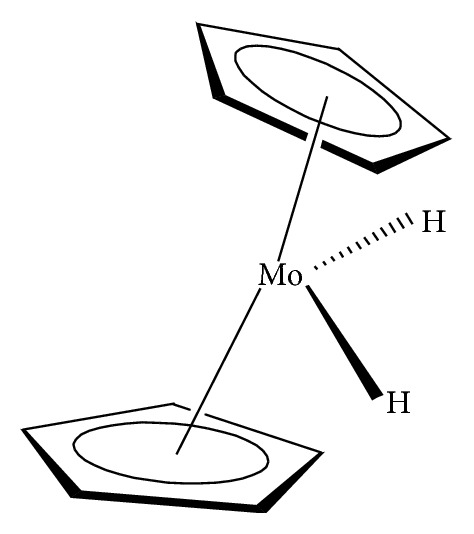
Molybdocene dihydride.

**Figure 5:**
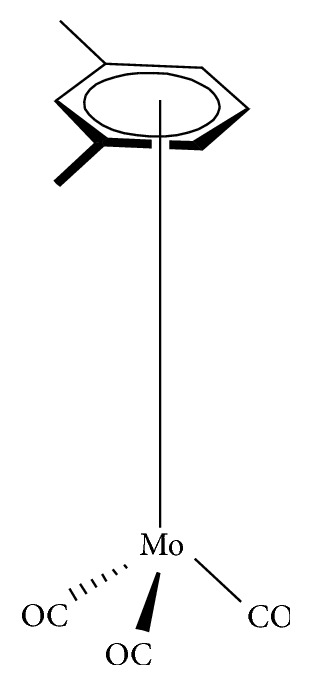
(Mesitylene)molybdenum tricarbonyl.

**Figure 6:**
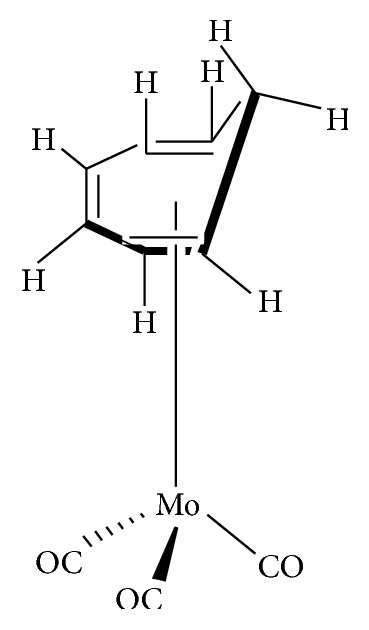
Cycloheptatrienenmolybdenum tricarbonyl.

Organomolybdenum compounds can be referred to coordinated molybdenum compounds in various oxidation states. They are potent anticancer and antimicrobial agents [8, 9, 17–19, 24–26]. According to Nair et al., biological applications of molybdenum complexes were due to the ability of incorporated ligands to chelate with trace metal ion (molybdenum ion), their rare action mechanisms, and capability to produce a high amount of harmful reactive oxygen species (ROS) which can interrupt the redox balance of a system leading to increase in deoxyribonucleic acid (DNA) damage, DNA protein cross-linked formation, lipid peroxidation, cellular toxicity, and/or wrong initiation of cellular signaling paths [8]. Results from their findings confirmed that Mo(V) had more cytotoxic activities than Mo(VI). On another note, Meléndez indicated that metallocenes are target specific drugs for cancer treatment. A metallocene (Figure 2) is an organometallic compound, which usually consists of two cyclopentadienyl anions (C_5_H_5_^−^, with Cp abbreviation) bound to a central metal (M) in the oxidation state, to yield (C_5_H_5_)_2_M [27]. Ndagi et al. [28] and Martin et al. [29] stated the lower attention on metallocenes (*molybdocene*, niobocene, vanadocene, and zirconocene) in perspective of cytotoxic impacts on cancer cell lines as compared to metal-based compounds. Marin et al. suggested extra optimization of these metallocenes for them to be used as anticancer agents in chemotherapy [29]. Molybdocene is a metallocene with a molybdenum atom. Molybdocene dichloride is the organomolybdenum which has the formula of (*η*^5^-C_5_H_5_)_2_MoCl_2_ (Figure 3). The International Union of Pure and Applied Chemistry (IUPAC) name is dichlorobismolybdenum(IV). Molybdocene dichloride had been reported to display anticancer activities, but there was a challenge of no yield of valuable compounds at the clinical stage [20]. Other organomolybdenum compounds are molybdocene dihydride, (mesitylene)molybdenum tricarbonyl, and cycloheptatrienenmolybdenum tricarbonyl, as shown in Figures 4–6, respectively.

## 3. Nanotechnology

The cancer disease with its proliferation has limited current chemotherapy. Nanotechnology in the form of nanomaterials makes available a possible alternative for cancer treatment [30–39]. Tran et al. examined the cytotoxicity of molybdenum trioxide (MoO_3_) nanoplates to disturbing breast cancer IMCF-7 cells by evaluating morphological variations and executing Western blot and flow cytometry analyses [40]. Their results proposed that MoO_3_ introduction encouraged apoptosis and produces reactive oxygen species (ROS) in IMCF-7 cells, thereby revealing the use of MoO_3_ for treating metastatic cancer cells in order to promote cancer therapy.

Molybdenum trioxide (molybdenum(VI) oxide; MoO_3_) nanoparticles were reported to be one of the metal nanoparticles to possess least toxicity [40, 41]. Apart from the roles molybdenum trioxide plays as an essential trace element, other roles are fuel cells [42], antimicrobial paints [43], potent antimicrobial agents [44], and membrane stress creation for microorganisms. Nanotechnology has transformed therapeutic approaches by improving the bioavailability, biodistribution, pharmacokinetics, stability, and targeted delivery to the required site, thereby decreasing toxicity, as well as reducing side effects [45, 46]. On the other hand, the main challenge encountered with the nanoparticles therapeutics is to overcome the improved permeability, retention effect, and targeted delivery to take full advantage of the effectiveness [46–49]. Among the nanotechnology carrier systems, electrospun nanofibres are developing to be a beneficial drug delivery system since they have massive packing capacity and targeted drug delivery [50, 51]. Recognition of an appropriate carrier system for the NPs would lessen various challenges. In this way, MoO_3_ could be used to fabricate a nanofibrous support with appropriate physicochemical properties to purposefully target cancer cells in order to overcome the challenges and lessen dosage and side effects [52–54].

For the past ten years, there had been an improved interest in the direction of low-dimensional nanostructured materials. Several efforts have been made to synthesize and apply one-dimensional (1D) nanomaterials based on transition metals [40, 55–58]. In this perspective, the 1D morphologies (for example, nanoflakes, nanorods, nanosheets, nanotubes, and nanowires) have been anticipated to display improved characteristics, making them appropriate for a wide range of uses, such as biofuel cells, bioimaging, *biosensors*, drug delivery, electrochromic devices, field emitters, light-emitting diodes, nanobioelectronics, nanogenerators, and supercapacitors [40, 55–62]. Recently, 1D nanostructured metal oxides in the direction of biosensors for cancer diagnostics aroused abundant interest [63]. Biosensors are used as optional technique for the most prevalent cancer, breast cancer, because it offers affordable cost, high sensitivity, least sample volume condition, and point-of-precaution diagnostics [64, 65]. Biomarkers have been recognised to perform a noticeable role as they are linked with the diagnosis and prognosis of a certain disease for biosensors development as points-of-care strategies [66, 67]. Numerous traditional tissue biomarkers, such as estrogen receptor (ER), progesterone receptor (PR), and human epithelial growth factor receptor-2 (HER-2), can be linked with the presence of breast cancer [68–70]. Among the three mentioned receptors, HER-2 is the only hopeful biomarker for breast cancer diagnosis [71, 72]. Human epithelial growth factor receptor-2 is a tyrosine kinase receptor which encrypts between 185 and 210 kDa protein located on chromosome 17 and is made up of cytoplasmic domain (CD), transmembrane domain (TD), and extracellular domain (ECD) [73–76]. It discharges its ECD into the serum section on above manifestation during the occurrence of breast cancer [77–81]. The limit concentration of HER-2 in serum sample of a breast cancer patient can be >15 *μ*g/mL [82–84]. As a result, monitoring HER-2 in serum can produce essential facts relating to tumour growth of a breast cancer patient. Recently, Gohring et al. established an optical-based biosensor based on optofluidic ring resonator for HER-2 detection [85].

Biomarkers perform important functions in the administration of patients with disturbing breast cancer [69, 86, 87]. Duffy et al. advocated that all laboratories assessing biomarkers for patient administration ought to use analytically and clinically certified assays, take part in external quality assurance programs, have recognized assay acceptance and rejection standards, implement regular audits, and be recognized by a suitable organization [69].

Weaver et al. stated that clinical breast care and breast cancer interrelated researches were influenced by imaging biomarkers [88]. They further stated that a previous incorporation of breast imaging with interrelated biomedical fields and the formation of large joint and shared databases of clinical, molecular, and imaging biomarkers would tolerate the field to continue controlling breast cancer care and research.

The aforementioned traditional biomarkers have various limitations, such as inability to arrest the spatial heterogeneity of breast cancer, and selected tissue cells during therapy might change the principal genotype and cause resistant treatment [89–91]. These can be overcome by molecular imaging biomarkers [86, 92].

**Figure 7:**
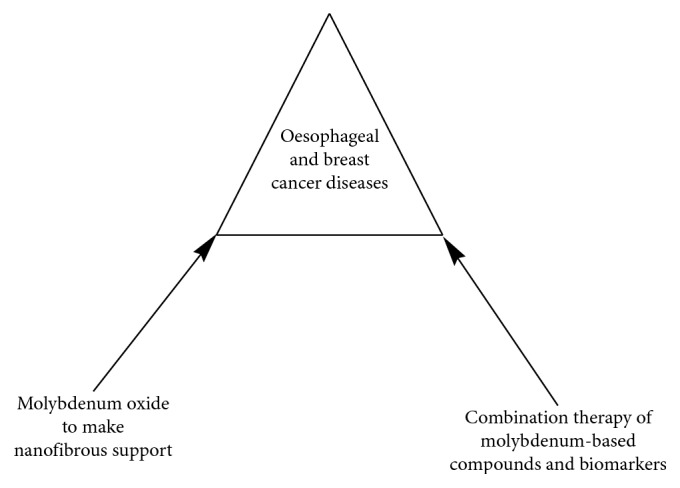
Use of molybdenum-based compounds as anticancer agent against oesophageal and breast cancer diseases.

The use of molybdenum-based compounds as anticancer agent against oesophageal and breast cancer diseases is shown in Figure 7.

### 3.1. Combination Therapy of Anticancer Agents and Biomarkers

According to Vivot et al., anticancer agents were progressively combined with a biomarker to decide if the prospective patient could benefit from the drug [93]. Two-thirds of Food and Drug Administration- (FDA-) endorsed anticancer agents require predictive biomarker testing to be based on clinical development restricted to biomarker-positive patients. From clinical evidence, they established that only limited cases of biomarker-negative patients would not benefit from treatment. They concluded that an absence of collective proof of clinical usefulness of biomarker testing for predictive biomarkers was identified as a challenge to precision medicine [93].

### 3.2. Combination Therapy of Metal-Based Nanoparticles (NPs) and Biomarkers

Metal-based nanoparticles (NPs) of various forms and magnitudes had been studied for their functions in diagnosis and the drug delivery system [28]. Combination of large drug dose is permissible on metal-based NPs because of the large surface area to volume ratio [28]. In order to increase the exactness in the diagnosis of cancers, Ndagi et al. studied different types of very accurate and very sensitive NP-based imaging platforms because these platforms are more advantageous compared with other agents [28]. These NPs can be functionalized to target accurately cancer and tumour cells, allowing the imaging and healing agents to be accurately delivered into these cells. Nanoparticles can be multifunctional. They show magnetic, optical, and structural properties which are deficient in a single molecule [28]. Ndagi et al. further stated that information on tumour-specific receptors, homing proteins, enzymes, and *biomarkers* are essential because tumour-specific targeting is attained by combining the NPs' surface with a molecule or *biomarker* attached to the tumour-cell receptor [28]. Synergistic effect can be attained by combining multifunctional NPs with different biomarkers and loading them with multidrug regimens, thereby decreasing the drug portion in the combination [94–96].

The use of nanostructured metal oxides and nanostructured transition metal oxides (nTMOs) to the development of an effective biosensing transform has motivated much interest [57]. In the midst of the nTMOs, nanostructured nMoO_3_ has been anticipated to have exceptional features, such as electrochemical activity, effective electrical properties, optical clearness, photochemical stability, and surface charge properties.

Augustine et al. established the constriction of label-free immunosensor biocompatible 1D nMoO_3_ biosensor for breast cancer biomarker detection [57]. Their results from electrochemical studies gave a wide linear detection range and excellent sensitivity. They ascribed the improved sensitivity of the biosensor to the mesoporous performance and high electrocatalytic activity of 1D MoO_3_, which offered high aspect ratio for enhanced bimolecular loading.

### 3.3. Selected Targets in Anticancer Drug Design and Molybdenum-Based Compounds

**Figure 8:**
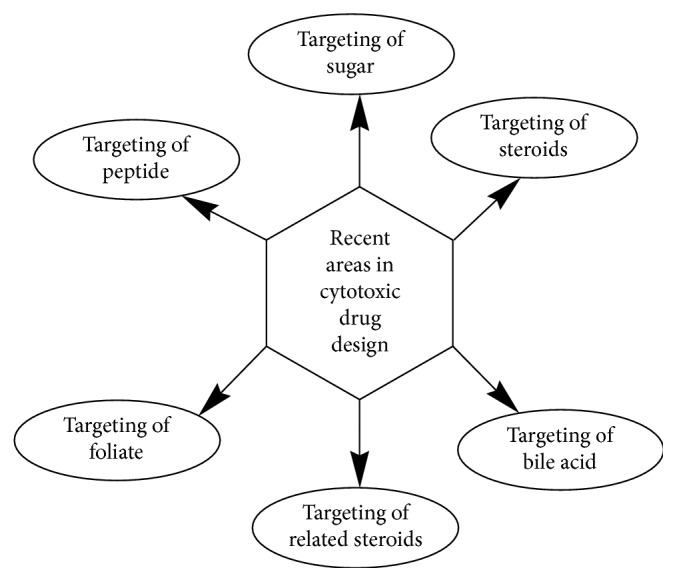
Selected targets in recent areas in cytotoxic drug design.

The notion of selecting targets brings hope in designing therapeutics which would selectively target cancer cells, leaving healthy cells intact. Molybdenum-based compounds with different functionalities can be developed and designed to have higher anticancer activities than cisplatin (platinum-based compound). The recent areas in cytotoxic drug design are targeting of sugar, targeting of steroids, targeting of bile acid, targeting of related steroids, targeting of folate, and targeting of peptide (Figure 8).

#### 3.3.1. Targeting of Sugar

Cancer cells need sugar (glucose) to survive. With this fact, biosugar facet could be used for drug targeting due to improved uptake of glucose by cancer cells [28, 97]. According to Johnstone et al.'s example, 2*αα*, 3-diaminosugars coordination compounds equivalent to oxaliplatin (functionalized cisplatin) were studied and discovered to have potential activity [28, 98]. Other prospective platinum-based compounds coordinated with glucose were also studied and discovered to have hopeful outcomes.

#### 3.3.2. Targeting for Steroid

Estrogen and testosterone are two of the several sex hormones which play important roles in drug targeting. They do this by combining steroidal units with nonliving group ligand. Ndagi et al. cited an example of estrogen receptor (ER), as a recognized drug target due to its extraordinary manifestation of protein on the faces of these cancer cells, which is predominantly, in breast cancer [28, 98]. Progress made in the research in this field led to the discovery of another conspicuous ER, labelled as ER*α*, while the previous ER was labelled as ER*β* [99]. Similarly, as estrogen targets platinum to the cancer cells displaying ER receptors, testosterone could target platinum to cancer cells displaying androgen receptor (AR) in order to increase deoxyribonucleic acid (DNA) and enhance the anticancer activity [28, 98].

#### 3.3.3. Targeting of Bile Acid

Bile acids are natural steroids and have been combined with platinum coordination compounds [28, 98]. For instance, a bile acid coordinated with dicarboxylate motif bound to a cisplatin piece was discovered to be an orally administered anticancer agent [28, 98].

#### 3.3.4. Targeting of Related Steroids

The translocator protein (TSPO) generally known as peripheral benzodiazepine regulates cholesterol transport and steroid syntheses [28, 100]. The protein is an essential target in cancer treatment due to its overexpression in various tumour cells [28, 100]. Ligated platinum(II) coordination compounds with bidentate thiazolylimidazopyridine were reported to interact intensely with the receptor [28, 100].

#### 3.3.5. Targeting of Folate

Folate is an essential carbon source for various cellular pathways, comprising DNA, ribonucleic acid (RNA), protein methylation, and DNA synthesis [101]. Improved folate uptake causes rapid cancer cell growth. This folate could be used as a baseline for drug targeting [101]. On another note, there is a control to the use of folate as a targeting agent of platinum complex. Previous study revealed that interaction of cisplatin with cellular folates would not be able to function as a cytosolic agent in a way similar to cisplatin [102]. In this regard, all researchers' hands are on deck to find the potential of folate in selecting drug targeting.

#### 3.3.6. Targeting of Peptide

Combination of platinum(II) complexes with the peptide results in the platination of complexes with anticancer activity [28, 101, 103–105]. Several platinum complexes combined with peptides have been screened against cancer cell lines, and a realistic number of them showed promising anticancer activities.

## 4. Conclusion and Future Direction

The use of molybdenum-based compounds as anticancer agents has been enhanced with the introduction of nanotechnology in the areas of nanofibrous support, combination therapy, and biomarkers. Selected targets are potentials in recent areas of anticancer drug design.

Future direction will entail the use of molybdenum-based compounds to substitute platinum coordination compounds in selecting targets in cytotoxic drug design.
